# Probiotics and triglyceride manipulation: potential implications for alleviating hypertriglyceridemia

**DOI:** 10.1016/j.jare.2025.06.036

**Published:** 2025-06-16

**Authors:** Mingliang Zhang, Weimin Jiang, Jie Yin, Dingfu Xiao

**Affiliations:** a College of Animal Science and Technology, Hunan Agriculture University, Changsha 410128, China; b Yuelushan Laboratory, Changsha 410128, China; c Hunan Institute of Animal and Veterinary Science, Changsha 410131, China

**Keywords:** Hypertriglyceridemia, Lowering triglycerides, Gut microbiota, Next-generation probiotics

## Abstract

**Background:**

Hypertriglyceridemia is one of the most prevalent lipid abnormalities encountered in clinical practice. Although many monogenic disorders causing severe hypertriglyceridemia have been identified, in most patients, triglyceride elevations result from a combination of multiple genetic variations with small effects and environmental factors. Common secondary causes include obesity, uncontrolled diabetes, alcohol abuse, high-fat foods, and the use of various commonly prescribed drugs. Notably, manipulating the gut microbiota through probiotic intervention may reduce triglyceride levels by targeting intestinal lipid absorption and host lipid metabolism.

**Aim of review:**

This paper reviews recent animal and human studies on probiotic interventions, summarizing the mechanisms through which probiotics reduce triglyceride levels and exploring the potential of next-generation probiotics in alleviating hypertriglyceridemia.

**Key scientific concepts of review:**

To discuss the link between gut microbiota and hypertriglyceridemia and the next generation of probiotics with the potential to reduce hypertriglyceridemia. Meanwhile, we also elucidate the mechanisms through which probiotics and their metabolites, such as short-chain fatty acids and bile acids, mediate multiple pathways to reduce triglyceride levels. Finally, the challenges and prospects of the application of probiotics were prospected.

## Highlights

• Summarizes the relationship between gut microbiota and hypertriglyceridemia.
• Discusses the potential mechanism by which probiotics reduce triglyceride levels.
• Prospects the challenges faced by probiotics and problems to be solved in the future.

## Graphical abstract

**Figure:**
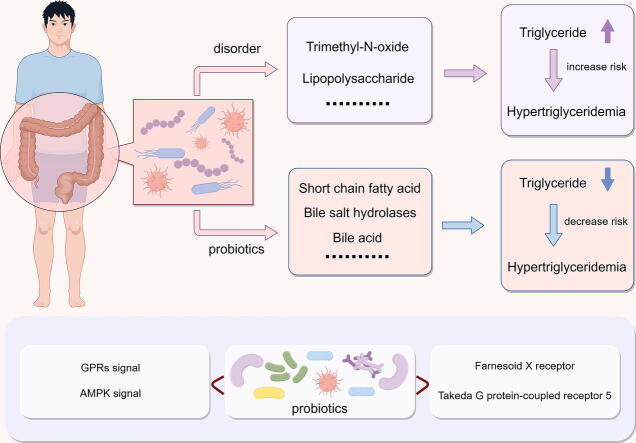

## Introduction

Hypertriglyceridemia (HTG) is a prevalent lipid abnormality observed in clinical practice characterizing by high fasting serum/plasma triglyceride (TG) levels equal to or exceeding 150 mg/dL (1.7 mmol/L) [1,2]. In recent years, the global prevalence of HTG has been increasing [1,3,4]. Specifically, in Western countries, 25 %–33 % of the adult population has HTG [3]. Notably, persistent HTG is related to high morbidity and mortality, primarily because of the increased risk of developing conditions such as acute pancreatitis, cardiovascular disease, non-alcoholic fatty liver disease (NAFLD), ischemic heart disease, and obesity [3,5].

In mammals, the majority of cell types possess the capability to synthesize TGs, with hepatocytes, intestinal mucosal cells, and adipocytes being the primary sites of TGs synthesis [6]. Notably, the ingested TG is hydrolyzed into monoglycerides and fatty acids with the assistance of lipase, then the lipids are emulsified by bile acids (BAs) to form micelles containing monoglycerides, fatty acids, cholesterol, and phospholipids [7]. Gut lipid transporting systems (i.e., CD36, fatty acid-binding proteins, and fatty acid transport proteins, and Niemann-Pick C1 like-1) further help to transfer the micelles into the enterocytes where lipids are re-esterified into TG and subsequently assembled into chylomicrons (CM) along with protein, phospholipid and cholesterol to enter the intestinal lymphatics [7,8]. Furthermore, the transport of TGs is also tightly regulated and optimized to balance their availability with the body’s energy demands [9]. To enable the transport of hydrophobic TGs within the aqueous extracellular medium of the body, TGs are encapsulated within the core of lipoproteins [2]. CM and very-low-density lipoproteins (VLDL) are the primary triglyceride-rich lipoproteins (TGRLs) synthesized by the intestine and liver, respectively, to transport dietary (exogenous) and endogenous lipids to peripheral tissues [2]. Particularly, dietary fat is primarily digested, absorbed, and transported in the small intestine, which contains a large number of microbial cells. Thus, manipulation of gut microbiota targeting intestinal lipid absorption may provide a potential for reducing TG levels and mitigating the risk of developing HTG through probiotics intervention.

In recent decades, advanced *meta*-omics research has pioneered novel pathways to elucidate the human microbiome contributes to improving human health, with most focus on HTG [[10], [11], [12], [13], [14], [15], [16]]. In a cohort study examining the relationship between antibiotic exposure and dyslipidemia in humans, exposure to enrofloxacin results in elevated TG levels, which correlates with a higher risk of HTG [17]. Furthermore, a separate study showed that transplanting the intestinal microbiota of humans with reduced TG levels into mice resulted in lower TG levels [18]. Notably, certain probiotics containing *Lactobacillus* and *Bifidobacterium* strains have been widely utilized to reduce TG levels in the host [19]. Together, the regulation of TG metabolism by gut microbiota suggests that interventions focusing on gut microbiota could effectively lower TG levels.

In recent years, the advanced sequencing methods and culturomics facilitate the identification of next-generation probiotics (NGPs), which are also referred to as live biotherapeutic products [10,20], including *Bacteroides*, *Akkermansia, Faecalibacterium, Lactococcus*, and butyrate-producing bacteria [10,21]. Despite the recognition of gut microbiota and the bacteria-derived metabolites in reducing TG levels, the specific mechanisms underlying the beneficial effects have not yet been reviewed in detail. Herein, we describe the potential of NGPs in reducing TG levels to alleviate HTG and summarize the current understanding of the molecular mechanisms through which the probiotic reduces TG levels.

## Gut microbiota and hypertriglyceridemia

Gut microbiota disorder-characterized by reduced microbial diversity, decreased abundance of beneficial bacteria, and increased abundance of potentially harmful bacteria-has been widely observed in HTG and its associated complications [11,16,22,23]. For example, a cohort study of elderly Danish adults found an association between gut microbiota dysbiosis and HTG, particularly with genus *Penicillium* and order Eurotiales being strongly associated with HTG [22]. Furthermore, HTG-associated acute pancreatitis was characterized by lower microbial diversity, with higher abundances of *Klebsiella*, *Escherichia/Shigella*, and *Enterococcus*, along with a decrease in beneficial bacteria, including *Faecalibacterium prausnitzii* and *Bacteroides uniformis* [11,23]. Interestingly, the study of 3432 Chinese individuals using bidirectional Mendelian randomization revealed that an increase in the relative abundance of *Oscillatoria* and *Alistella* in stool was causally associated with a reduction in TG concentrations [24]. Thus, governing gut microbiota communities may help improve or re-establish gut microbiota, modulate host lipid metabolism, and reduce TG levels, thereby alleviating HTG. Conventional probiotics have demonstrated efficacy in reducing TG levels; however, their sustained colonization within the human gastrointestinal tract exhibits significant variability [25]. Conversely, NGPs strains exhibit a robust capacity for effective gut colonization, potentially exerting beneficial health effects through mechanisms distinct from those associated with conventional probiotics [10]. Here, we summarized the roles and findings related to NGPs strains in the reduction of TG levels, thereby offering a reference for the application of NGPs.

### Akkermansia muciniphila

*Akkermansia muciniphila*, part of the mucus-layer-gut microbiota, occupies a distinct niche in the human gut and is also a prominent focus in the emerging research field of NGPs [[26], [27], [28]]. Studies have demonstrated that *Akkermansia muciniphila* significantly contributes to host health improvement, particularly in the regulation of lipid metabolism. For example, oral *Akkermansia muciniphila* significantly reduces serum TG levels in obese mice [29]. The TG-lowering effect is further validated in a study involving overweight or obese insulin-resistant individuals, with a 20.05 % reduction in TG levels observed after oral live *Akkermansia muciniphila* [30]. In addition, oral *Akkermansia muciniphila* improves insulin sensitivity and liver function while reducing inflammation [30]. Interestingly, the overall gut microbiota structure is unaffected [30].

The current data indicate that *Akkermansia muciniphila* influences TG regulation through various pathways, including short-chain fatty acids (SCFAs), BAs, and secreted proteins. Notably, the P9 protein secreted by *Akkermansia muciniphila* is capable of inducing the release of glucagon-like peptide-1 (GLP-1) and promoting thermogenesis in brown adipose tissue [31]. In addition, Amuc_1100, a membrane protein secreted by *Akkermansia muciniphila*, mitigates high-fat diet (HFD)-induced adipocyte hypertrophy and alleviates obesity [28]. Therefore, *Akkermansia muciniphila* holds significant potential for reducing TG levels.

### *Bacteroides* SPP

The gut is colonized with abundant genera of *Bacteroides*, most of which are associated with host lipid metabolism and have been shown to modulate TG levels in animal models. For instance, *Bacteroides uniformis* has been shown to ameliorate immune dysfunction and reduce serum TG levels in HFD-induced mice [32]. Notably, patients with HTG-associated pancreatitis have a lower abundance of *Bacteroides uniformis* [11]. Additionally, *Bacteroides vulgatus* and *Bacteroides ovatus* can reduce TG levels in rat models of hyperlipidemia [33] and mouse models of NAFLD [34], respectively, thereby improving host health. Therefore, these *Bacteroides* is a promising probiotic candidate that may help reduce TG levels.

However, *Bacteroides fragilis* has also been shown to exacerbate NAFLD induced by a HFD, resulting in lipid accumulation [35]. Additionally, *Bacteroides stercoris* shows a positive correlation with intrahepatic TG content [18]. These data suggest that the safety of specific strains within the genus *Bacteroides* should be assessed, particularly with regard to their potential to reduce TG levels.

### Clostridium butyricum

*Clostridium butyricum*, part of Clostridium group I, is gram-positive obligate anaerobic bacterium and commonly present in gut of animals and humans [36]. In recent years, the beneficial or protective effects of *Clostridium butyricum* on human diseases have been extensively reported [36]. For instance, in mice fed HFD, dietary supplementation with 7 % *Clostridium butyricum* reduces serum TG levels and increases LDL-C levels [37]. Moreover, a clinical study found that *Clostridium butyricum* significantly decreases TG levels in patients suffering from nonalcoholic fatty liver disease [38]. Currently, certain strains of *Clostridium butyricum* are commercially available as over-the-counter probiotics [10], frequently utilized to improve gut health and manage diarrhea [36]. Notably, *Clostridium butyricum* plays a crucial role in regulating host lipid metabolism, suggesting that these strains may be beneficial for lowering TG levels.

### *Eubacterium* SPP.

*Eubacterium* spp., a core genus of the human gut microbiome, is widely distributed across the human population [39]. Several members of the genus *Eubacterium* play key roles in lipid metabolism, immune regulation, and suppression of inflammation. For example, a study involving 65 Mexican participants (38 obese and 27 controls) found that *Eubacterium hallii* is negatively correlated with TG levels [40]. Notably, the role of *Eubacterium* spp. in TG is further validated in a mouse study that oral *Eubacterium hallii* significantly reduces hepatic TG levels and improves insulin sensitivity [41], suggesting a potential positive effect in TG manipulation.

### Faecalibacterium prausnitzii

*Faecalibacterium prausnitzii* is a key microbiota component and biosensor in human gut health and is among the most common bacterial species in the colon [42]. Notably, patients suffering from type 2 diabetes and obesity exhibited an inverse correlation with *Faecalibacterium prausnitzii* [43]. *Faecalibacterium prausnitzii* has been found to significantly reduce TG levels [[43], [44], [45]] and inflammation in fat tissue in mice fed a HFD [44]. Interestingly, *Faecalibacterium prausnitzii* also suppresses appetite, modulates gut microbiota, and enhances levels of SCFAs [[43], [44], [45]]. Additionally, *Faecalibacterium prausnitzii* has been demonstrated to lower TG levels in mice suffering from type 2 diabetes [46]. These results highlight the important role of *Faecalibacterium prausnitzii* in reducing TG levels. Hence, *Faecalibacterium prausnitzii* holds a potential positive effect in TG manipulation.

### *Parabacteroides* SPP.

*Parabacteroides distasonis* and *Parabacteroides goldsteinii* from the genus *Parabacteroides* are considered potential NGPs [47]. The health-promoting and host-beneficial attributes of *Parabacteroides distasonis*, a key part of the human gut microbiota, have recently drawn significant attention, highlighting its potential as a novel biological therapeutic agent [48]. For instance, in mice fed a HFD, *Parabacteroides distasonis* reduces serum TG, LDL-C, and free fatty acids [49]. The mechanism may be associated with succinic acid and secondary BAs derived from *Parabacteroides distasonis*, which activate gluconeogenesis and the farnesoid X receptor (FXR) pathway, thereby alleviating obesity and metabolic dysfunction [50].

In addition, *Parabacteroides goldsteinii* is a significant constituent of the human intestinal microbiota and is closely linked to improving host lipid metabolism [47,51]. In an experiment involving *Helicobacter pylori*-infected mice, *Parabacteroides goldsteinii* MTS01 significantly reduces TG levels and alleviates systemic inflammation [51]. This may be related to increasing adipose tissue heat production, enhancing intestinal integrity, and reducing insulin resistance, mediated by *Parabacteroides goldsteinii* [52].

## Triglyceride-lowering mechanisms of the next-generation probiotics

The gut microbiota, including probiotics, face challenges in crossing the gut barrier [53,54], thereby limiting the direct impact on the synthesis and catabolism of TGs. However, the gut barrier is selectively permeable, allowing the translocation of microbial metabolites from the intestinal lumen into the circulation [53,54]. Consequently, the effects of probiotics are primarily mediated through the metabolites. Interestingly, among these metabolites, the levels of trimethyl-N-oxide (TMAO), lipopolysaccharide (LPS), SCFAs, and BAs are likely closely associated with TG metabolism. Specifically, TMAO enhances BAs synthesis, alters liver BAs composition to promote FXR antagonistic activity, and accelerates TG synthesis by inhibiting the liver FXR signaling pathway [55]. Additionally, in vitro experiments have shown that LPS inhibits fatty acid oxidation (FAO), resulting in elevated TG levels [56]. In contrast, SCFAs and BAs produced by gut microbiota are beneficial for host metabolic health and help reduce TG levels by regulating lipid metabolism and immune homeostasis [57,58]. The diversity in these mechanisms highlights the complexity of host-strain interactions and underscores the need for further research to elucidate the specific pathways involved in TG reduction across different strains. Hence, a comprehensive summary of the TG-lowering mechanisms of microbial metabolites and other related molecules could enhance the identification and utilization of microbes with the potential to reduce TG levels, thereby advancing the development of novel microorganisms with clinical applications.

### Short-chain fatty acids

SCFAs are mainly generated by the gut microbiota in the colon and show a gradient distribution throughout the body [59]. Interestingly, acetate and butyrate have also been produced in the small intestine [60]. For example, *Clostridium butyricum*, *Eubacterium*, *Faecalibacterium prausnitzii*, *and Ruminococcus bromii* are the main bacteria responsible for butyrate production in the human gut [10,59]. Other bacteria such as *Butyricimonas*, *Bifidobacterium, Dysosmobacter*, and *Subdoligranulum* can also produce SCFAs [10,36,61]. *Bacteroides*, a primary SCFAs producer in the gut, shows a positive link between SCFA levels in fecal matter [10,62]. Notably, a study highlighted that in mice fed a HFD, SCFA supplementation led to a significant decrease in hepatic TG levels, pointing to SCFAs as effective molecules for TG level reduction [57]. Collectively, these findings illustrate that diverse gut microbiota have the ability to generate SCFAs, which may contribute to the reduction in TG levels.

#### Short-chain fatty acids-GPRs signal

**Fig. 1:**
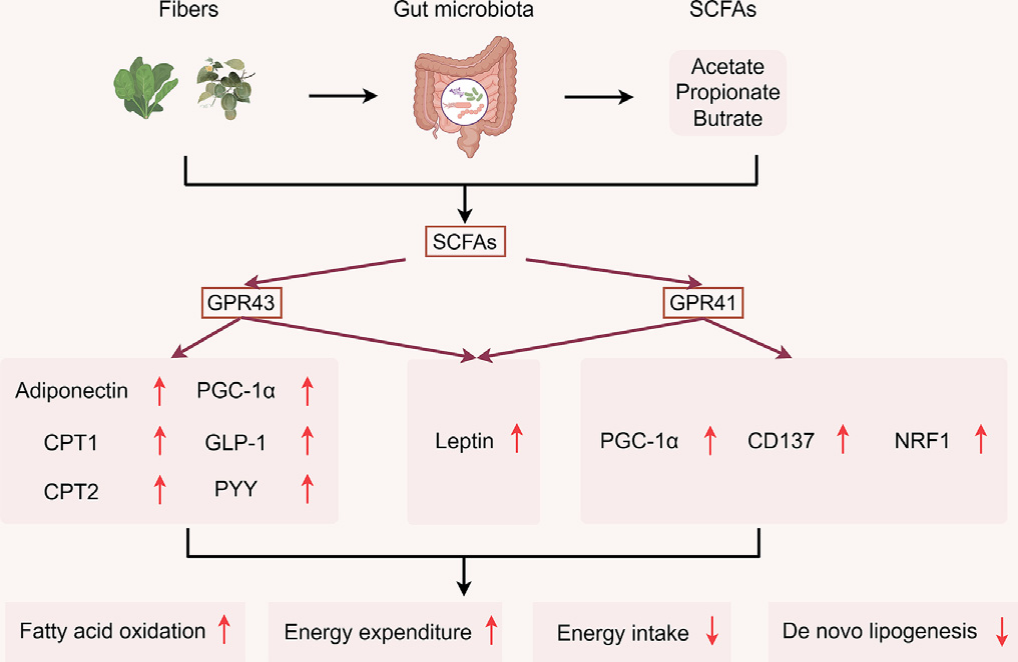
Proposed mechanisms by which short-chain fatty acids activate GPR43/41 to reduce triglyceride levels.

SCFAs may reduce TG levels in the host through following pathways (Fig. 1): as signaling molecules, SCFAs bind to and activate the orphan G protein-coupled receptors 43 and 41 (GPR43 and GPR41), also known as free fatty acid receptors 2 and 3 (FFAR2 and FFAR3), thereby sensing nutrient status and transduces signals that inhibit lipid accumulation, ultimately lowering TG levels and preserving cellular homeostasis [57,[63], [64], [65], [66]]. Specifically, GPR43 is positively correlated with a variety of metabolism-related factors, including adiponectin, carnitine palmitoyltransferase1/2 (CPT1/2), peroxisome proliferator-activated receptor-γ coactivator-1α (PGC-1α), cytochrome *c* oxidase IV (COX IV), β-F1-ATPase, GLP-1, and peptide YY (PYY) [63]. In addition, GPR41 is positively correlated with PGC-1α, CD137, and nuclear respiratory factor 1 (NRF1) [63]. Notably, in adipocytes, SCFAs-GPRs interactions lead to increased leptin production [67], a signaling molecule that stimulates hepatic FAO and inhibits de novo lipogenesis (DNL) through phosphorylation of acetyl-CoA carboxylase 1 (ACC1) [68]. These data suggest that SCFAs enhance FAO, energy expenditure and regulate the secretion of hormones related to satiety by activating GPR43 and GPR41, potentially contributing to the reduction of TG levels [57,63,65,66,69].

#### Short-chain fatty acids-AMPK signal

**Fig. 2:**
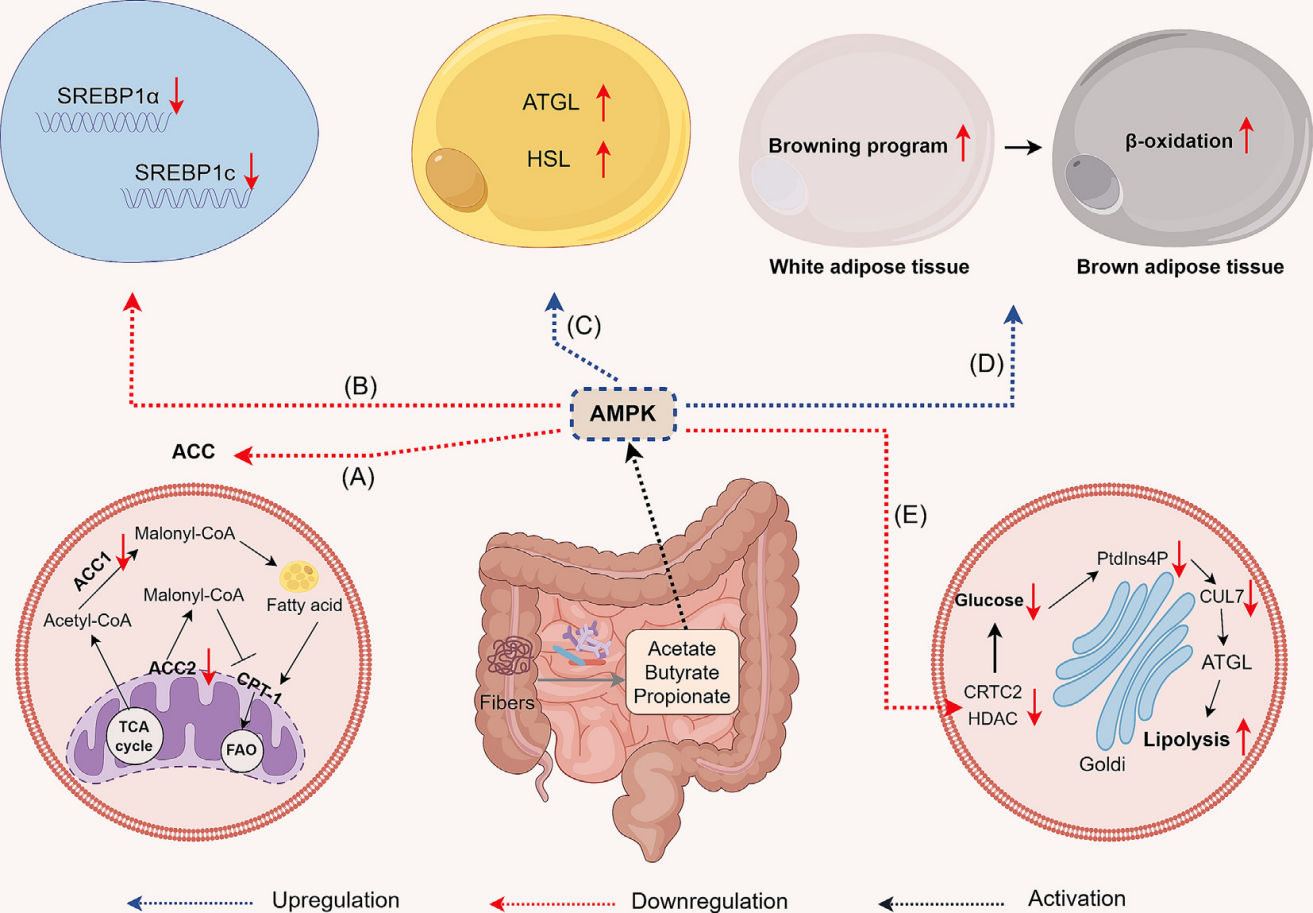
Proposed mechanisms by which SCFAs activate AMPK to reduce triglyceride levels. (A) SCFAs suppress ACC activity through the activation of AMPK, consequently inhibiting DNL and promoting FAO. (B) SCFAs inhibit the expression of SREBPs through AMPK activation. (C) SCFAs activate AMPK and enhance lipase activity. (D) SCFAs enhance β-oxidation and promote adipocyte browning by activating the β3-adrenergic receptor (β3-AR)/AMPKα signaling pathway. (E) SCFAs promote AMPK phosphorylation and inhibit transcription factors that regulate the activation of glycolysis and lipogenesis transcriptional programs.

Various studies have proven that SCFAs are able to activate the AMPK signaling pathway [[70], [71], [72]]. Notably, the role of AMPK in TG metabolism regulation can be categorized into two primary functions, inhibition of anabolic and stimulation of catabolic [[73], [74], [75], [76]]. Here, we elucidate the potential mechanism by which SCFAs reduce TG levels via AMPK activation (Fig. 2).

SCFAs suppress ACC activity through the activation of AMPK, consequently inhibiting DNL and promoting FAO, which contribute to reduce TG levels [[75], [76], [77]]. ACC is present in two forms: ACC1, mainly found in the cytosol and involved in fatty acid biosynthesis, and ACC2, linked to the outer mitochondrial membrane and controlling fatty acid β-oxidation [[75], [76], [77], [78]]. ACC is a crucial enzyme in fatty acid metabolism, responsible for catalyzing the ATP-dependent conversion of acetyl-CoA to malonyl-CoA. This reaction is critical as malonyl-CoA acts as an inhibitor of Carnitine Palmitoyltransferase 1 (CPT1), the enzyme responsible for the transport of long-chain fatty acids into mitochondria for subsequent oxidation [68,[75], [76], [77], [78]]. AMPK modulates cellular lipid metabolism by phosphorylating ACC1 at Ser79 and ACC2 at the orthologous site Ser212, inhibiting fatty acid synthesis, and promoting FAO [77,79]. This process alleviates the inhibition of CPT1 by malonyl-CoA, which is generated by ACC2 at the mitochondrial outer membrane [77,79]. In vitro experiments demonstrated that acetic acid activates the AMPKα signaling pathway and inhibits acetyl-CoA carboxylase activity in bovine hepatocytes, decreasing TG levels [80] (Fig. 2A).

AMPK phosphorylates and inhibits transcription factors like sterol regulatory element-binding proteins (SREBPs), which are crucial for activating glycolytic and lipogenic transcriptional programs and regulating lipid synthesis [58,77]. Specifically, SREBP1α is involved in lipid synthesis and growth, while SREBP1c is associated with the synthesis of fatty acids and the storage of energy [81]. Notably, acetate has been demonstrated to down-regulate the expression of SREBP1 and decrease serum TG levels in rat [82] and pig [83]. Furthermore, other studies have reported that adiponectin activates the liver kinase B (LKB)-AMPK pathway via adipoR1, which inhibits SREBP1c expression and subsequently reduces liver adipogenesis and the expression of related genes [68,84] (Fig. 2B). AMPK phosphorylates lipases, including hormone-sensitive lipase (HSL) and adipocyte triglyceride lipase (ATGL), to regulate lipolysis and FAO in adipose tissue, thereby reducing TG levels [74,77,85]. Furthermore, studies conducted on mice and cellular models have demonstrated that the activation of AMPK leads to an upregulation in the activity of ATGL and HSL [78,86,87] (Fig. 2C).

SCFAs enhance β-oxidation and promote adipocyte browning by activating the β3-adrenergic receptor (β3-AR)/AMPKα signaling pathway, thereby inhibiting lipid accumulation in adipocytes [88]. β3-AR and AMPKα have been identified as key regulators in stimulating the browning program of white adipose tissue [88]. In vitro experiments, SCFAs increased mitochondrial number in 3T3-L1 adipocytes and upregulated the expression of fatty acid β-oxidation marker genes (*PPARα*, *CPT1α*, *ACOX1*) [88]. However, treatment of 3T3-L1 adipocytes with β3-AR and AMPK antagonists decreased the expression of brown adipose-specific proteins (UCP-1 and PGC-1α), further indicating that SCFAs activate β3-AR/AMPK to reduce TG levels [88] (Fig. 2D).

AMPK suppresses the transcriptional induction of gluconeogenesis, the process of de novo synthesis of glucose, by phosphorylating and nuclear exclusion of cyclic AMP-regulated transcriptional co-activator 2 (CRTC2) and class II histone deacetylases (HDACs), which are essential co-factors for the transcription of gluconeogenic genes [77]. However, the coordination between glucose and fatty acid supply is particularly important in meeting various energy needs [77]. Recent research has demonstrated that Golgi PtdIns4P plays a regulatory role in ATGL-mediated lipolysis through the mechanism of intracellular glucose sensing. Specifically, depletion of intracellular glucose leads to a reduction in Golgi phosphatidylinositol 4-phosphate (PtdIns4P) levels, consequently diminishing the assembly of the E3 ligase complex CUL7^FBXW8^ within the Golgi apparatus [89]. Furthermore, this decrease in the E3 ligase complex subsequently results in reduced polyubiquitylation of ATGL in the Golgi, thereby enhancing ATGL-mediated lipolysis [89]. In summary, AMPK phosphorylation inhibits CRTC2 and HDACs, thereby suppressing gluconeogenesis and reducing the glucose levels. This may represent an intrinsic pathway mediated by a glucose-sensing mechanism that involves the Golgi PtdIns4P-ATGL-lipolysis pathway [89], which promotes lipolysis and enhances the release of fatty acids from lipid droplet stores in response to glucose depletion (Fig. 2E).

### Bile salt hydrolases

In the gut, bile salt hydrolases (BSHs) break down amide bonds in conjugated BAs, which enhances the variety of the bile acid pool [90]. Notably, BSH protein sequences were identified in 591 intestinal bacterial strains spanning 117 genera within the human microbiota, with 27.52 % of these strains possessing BSH paralogs [91]. The unconjugated BAs generated by BSHs act as signaling molecules that not only control BAs metabolism and transport but also significantly influence lipid metabolism, insulin sensitivity, and innate immunity [10,90]. The role of BSHs in lowering cholesterol levels in the host is significant and may also possess the capacity to reduce TG levels. Within the small intestine, micelles are formed by the interaction of BAs with fatty acids and monoglycerides [10]. However, BSHs generate uncoupled BAs that are more hydrophobic and less soluble compared to coupled BAs, which decreases their passive transport into intestinal cells [10]. Consequently, increased BSHs activity may decrease lipid absorption, thereby promoting TG-lowering effects. Notably, oral administration of Lactobacillus plantarum Y15 with high BSHs activity reduce TG levels in obese mice [92]. Furthermore, *Escherichia coli* MG1655 (a K-12 strain that lacks BSH activity) expressing the BSH gene significantly reduced liver TG levels in mice fed either normal or HFD [93]. This may be due to bacterial BSH deconjugating the FXR antagonist tauro-β-MCA, which facilitates the inhibition of adipogenesis by liver FXR [93,94].

### Bile acids

In the liver, cholesterol undergoes a series of enzymatic processes to synthesize BAs, primarily resulting in primary BAs like cholic acid (CA) and chenodeoxycholic acid (CDCA) [95]. Gut bacteria convert primary BAs into secondary BAs through 7-α-dehydroxylation, primarily resulting in deoxycholic acid (DCA) and lithocholic acid (LCA) [[94], [95], [96], [97], [98]]. Hence, BAs represent a critical class of metabolites produced by gut microbiota. Recent research has demonstrated that a diverse array of gut bacteria, such as species from the genera *Bacteroides*, *Lactobacillus*, *Bifidobacterium*, *Enterobacter*, *Ruminococcus*, *Eubacterium*, and *Clostridium*, are crucial in bile acid metabolism [97,98]. Furthermore, BAs act as signaling molecules that modulate BA biosynthesis as well as lipid and glucose homeostasis, energy expenditure, and immune signaling [[94], [95], [96], [97], [98]]. BAs not only mediate cholesterol metabolism [10], but also regulate TG homeostasis [58,99]. Notably, CA inhibited hepatic TG accumulation, and VLDL secretion in a mouse model of HTG [58]. In summary, BAs are integral to the regulation of lipid homeostasis and may contribute to the reduction of TG levels.

#### Bile acids-farnesoid X receptor

**Fig. 3:**
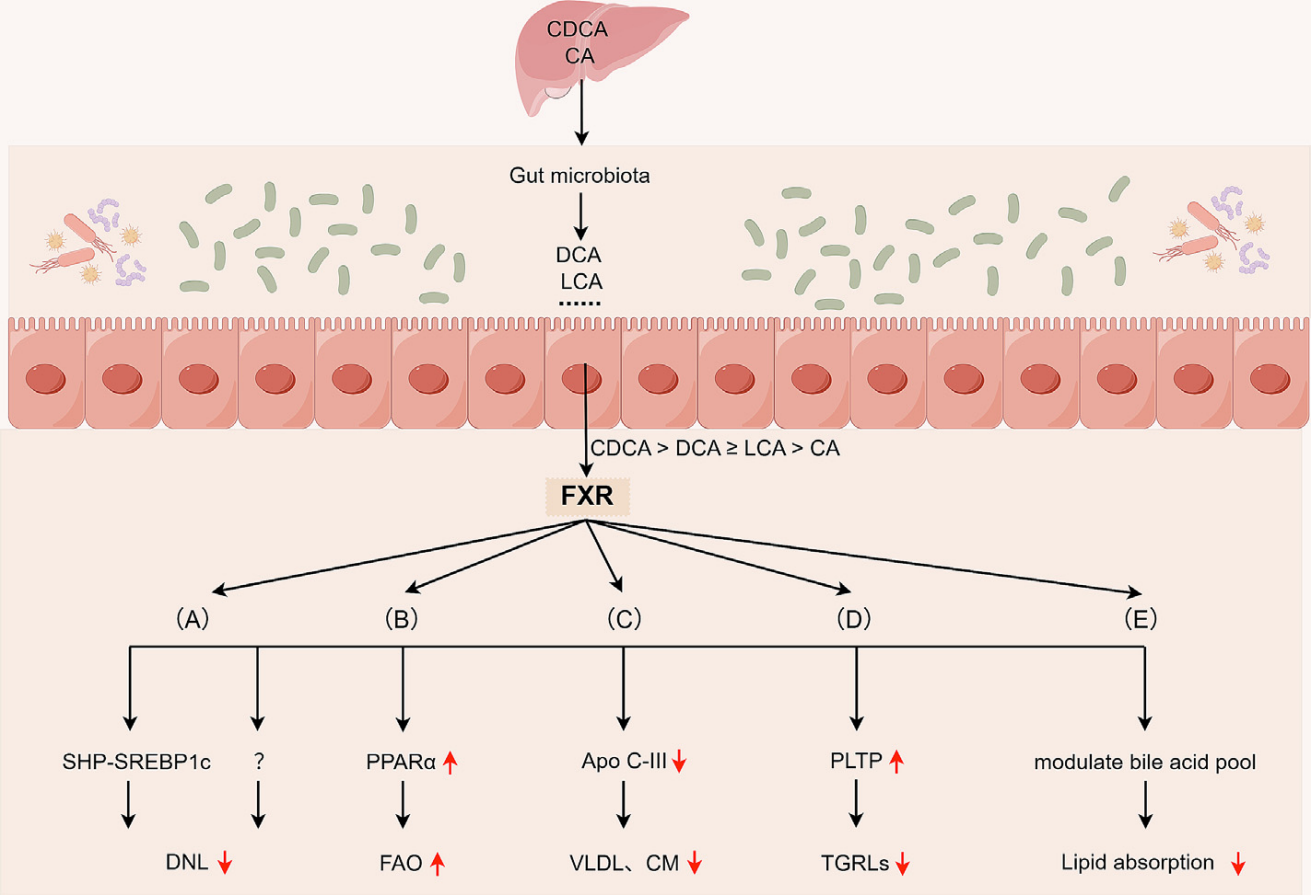
BAs activate FXR and regulate key enzymes, lipoproteins, and their receptors involved in the triglyceride metabolic pathway, thereby reducing triglyceride levels.

FXR functions as a bile acid-regulated transcription factor and protective sensor, is endogenously activated by BAs [94,100]. Notably, the binding affinity of the BAs mediating FXR stimulation was ranked in descending order of strength: CDCA > DCA ≥ LCA > CA [10,101]. FXR modulates TG metabolism by regulating essential enzymes, lipoproteins, and their associated receptors involved in TG metabolic pathways (Fig. 3).

The mechanism by which BAs activate FXR to lower TGs is to reduce DNL via the FXR-short heterodimer partner (SHP)-SREBP1c pathway [58] (Fig. 3A). In addition to mediating the FXR-SHP-SREBP1C pathway, FXR suppresses DNL genes (including Steroyl Coa Desaturase 1, Lipin 1 and Acyl Coa: Diacylgycerol Acyltransferase 2) through an unknown mechanism [100] (Fig. 3A). Activation of FXR upregulate the expression of PPARα, enhance the expression of enzymes involved in the FAO pathway, and facilitate the mitochondrial β-oxidation of fatty acids [101,102] (Fig. 3B). In vitro study has shown that cells overexpressing FXR show increased transcription of genes involved in FAO signaling pathways and mitigate intracellular lipid accumulation [102]. Another study demonstrated that dietary supplementation with hyodeoxycholic acid reduced hepatic TG levels in mice with NAFLD by promoting FAO through PPARα-dependent mechanism [103].

FXR activation by both natural agonist CDCA or taurocholic acid suppresses hepatic Apo C-III expression [101] (Fig. 3C). Notably, Apo C-III, predominantly synthesized by hepatocytes (comprising 80–90 % of its production), constitutes a significant component of VLDL and CM [104]. Furthermore, Apo C-III is vital for inhibiting lipoprotein lipase and non-lipoprotein lipase [104]. Thus, FXR can effectively inhibit the accumulation of TGs by inhibiting Apo C-III expression.

FXR enhances the expression of phospholipid transfer protein (PLTP), thereby reducing plasma TG levels [105] (Fig. 3D). Specifically, PLTP is crucial for the exchange and transport of soluble substances within the liver, especially exhibiting a high affinity for phospholipids [105,106]. Upon activation by FXR, PLTP facilitates the transfer of phospholipids and cholesterol from TGRLs (CM and VLDL) to HDL, thereby sustaining HDL levels in plasma and contributing to the overall reduction of plasma TG levels [105]. Notably, exogenous administration of 1 % cholic acid significantly upregulated *PLTP* gene expression in the liver of mice [105]. FXR modulates the composition of the bile acid pool, particularly by reducing BAs, inhibiting intestinal lipid absorption, and decreasing hepatic TG accumulation [100] (Fig. 3E). Overall, the activation of FXR inhibits the synthesis of endogenous fatty acids and TGs while promoting FAO, thereby reducing TG levels.

#### Bile acids-Takeda G protein-coupled receptor 5

**Fig. 4:**
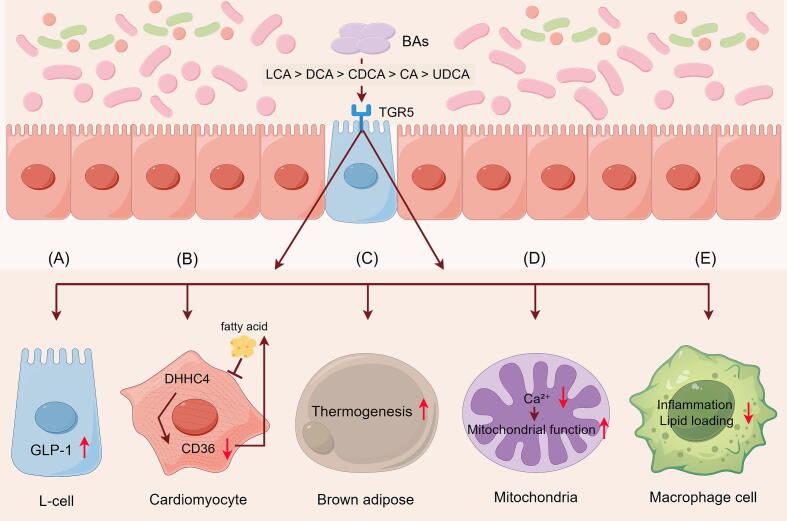
Proposed mechanisms by which BAs activate TGR5 to reduce triglyceride levels. (A) BAs activate TGR5, promoting the secretion of GLP-1 from intestinal L cells, thereby inhibiting lipogenesis. (B) BAs activate TGR5, inhibit CD36 palmitoylation, and consequently reduce fatty acid uptake. (C) BAs activate TGR5, thereby enhancing the energy expenditure of brown adipose tissue. (D) BAs activate TGR5 and alleviate mitochondrial dysfunction caused by Ca^2+^ overload. (E) BAs activate TGR5, reducing macrophage inflammation and lipid accumulation.

Takeda G protein-coupled receptor 5 (TGR5), also referred to as GPBAR1, M-BAR, and BG37, belongs to the family of G protein-coupled receptors [107,108]. Notably, extensively expressed in multiple regions of the human body, TGR5 is important for the regulation of metabolic processes, including lipid metabolism, weight management, and the maintenance of blood glucose homeostasis [107,108]. Furthermore, the agonistic effects on TGR5 differed depending on the type of BA, with potency ranking as follows: LCA > DCA > CDCA > CA > UDCA [107]. Here, we summarize the potential mechanism by which BAs mediate TGR5 to facilitate the reduction of TG levels (Fig. 4).

TGR5 activation has the potential to cause intestinal endocrine L cells to secrete GLP-1, which subsequently circulates to pancreatic β-cells to promote insulin secretion [94,107] (Fig. 4A). Research indicates that GLP-1 can inhibit hepatic lipogenesis [107] and reduce the synthesis rate of TGRLs-apob-48 in the intestinal [109]. In a study with mice fed a HFD, activating the GLP-1 receptor led to decreased VLDL particle production and downregulated hepatic lipogenesis-related gene expression (*Srebp-1c*, *Fasn*, *Dgat1*) [110]. Additionally, a clinical study found an association between impaired GLP-1 secretion and HTG [111]. These findings indicate that the GLP-1 signaling pathway, activated via TGR5, may serve as a therapeutic target for reducing TG levels.

TGR5 activation may reduce lipid accumulation by regulating CD36, a critical fatty acid transporter, to inhibit fatty acid uptake [108] (Fig. 4B). Mechanistically, TGR5 inhibits the palmitoylation of CD36 via DHHC4, preventing its localization to the plasma membrane and thereby reducing fatty acid uptake and lipid accumulation in cardiomyocytes [108]. The enhancement of TGR5-mediated thermogenesis in brown adipose tissue by BAs signaling results in decreased hepatic uptake of free fatty acids and reduced lipid accumulation [112] (Fig. 4C). In mouse studies, it was confirmed that treatment with the TGR5-specific agonist 23(S)-methyl-lithocholic acid promoted BAT activation and reduce TG levels [112].

TGR5 is a critical regulator of mitochondrial function [113]. TGR5 activation alleviates mitochondrial dysfunction caused by Ca^2+^ overload [113], thereby enhancing FAO. Hence, TGR5 not only improves mitochondrial function but may also contribute to the reduction in TG levels (Fig. 4D). Interestingly, there is a strong association between elevated TG levels and atherosclerosis [114], and activating TGR5 can inhibit atherosclerosis by minimizing inflammation in macrophages and lipid loading [115]. Therefore, TGR5 may reduce TG levels by inhibiting macrophage inflammation (Fig. 4E). In conclusion, TGR5 can be activated via multiple mechanisms and holds potential for the reduction of TG levels.

### Bacteria-lowering triglycerides

In addition to interacting with the host to lower TGs levels, microbes themselves possess the potential to reduce TGs levels. For example, in an in vitro experiment, *bifidobacteria* strains inoculated in an MRS-TG medium for 72 h reduced the TG content in the supernatant [116]. Similarly, lactic acid bacteria such as *Lactobacillus rhamnosus* S51, *Lactobacillus plantarum* S184, and *Lactobacillus fermentum* S7 can reduce TG levels in vitro [117]. Interestingly, bacteria may also reduce TGs through emulsification and utilization of grease [118]. These findings imply that microbes themselves have a TG-lowering effect. The mechanism may involve bacterial consumption of TG, which reduces TG contents in the intestine, thereby decreasing the body's TG absorption. However, the precise changes remain unclear and further research is required to identify the key mechanisms by which microorganisms reduce TG levels in vitro.

## Conclusions and perspectives

In summary, HTG, characterized by elevated TG levels, is associated with increased high morbidity and mortality [3,5]. Therefore, reducing TG levels is of significant clinical importance in the treatment of HTG and its associated complications. However, the use of TG-lowering drugs such as statins, niacin, and omega-3 polyunsaturated fatty acids (PUFAs), which exhibit a broad range of effects, may have limited efficacy [9]. Hence, the development of new drugs and the pursuit of alternative drugs are particularly important. Notably, microbes have been demonstrated to beneficially lower host TG levels. In this review, we discuss the link between gut microbiota and HTG, and explore the potential of NGPs in TG reduction. We also summarize the probiotics mediation of TG reduction through various pathways, including SCFAs and BAs. These insights will help identify new microbes and beneficial metabolites, further advancing the transition from conventional to precision probiotics. However, several issues remain to be addressed and clarified in the future. For example, how can the NGPs be efficiently and accurately screened? We believe that, in the future, the NGPs can be screened at high throughput using genomics big data analysis and AI-based machine learning algorithms. Additionally, biosensors, engineered through genetic modification, can be employed to identify specific strains that respond to target metabolites. Microbial-host interactions are intricate and multifaceted, and how can genetic engineering be applied to enhance the safety of NGPs? Additionally, the quality standards for probiotic preparations are not yet optimal. How can strict quality control standards for microbial preparations be established to ensure the safety and efficacy of these products? Differences exist in the structure of the gut microbiome across individuals, which may lead to variability in the effectiveness of microbial interventions. How can a personalized probiotic intervention program be developed based on the unique microbial characteristics of each individual? In recent years, the rhythms of gut microbiota and the functions of the host's central and peripheral circadian clocks have been established [119,120], while the circadian rhythms and microbial effects on gut lipid absorption remain underexplored. Hence, further elucidation of the microbial rhythms may hold significant clinical value for the precise application of probiotics.

## Compliance with ethics requirements

This article does not contain any studies with human or animal subjects.

## Credit author statement

All authors have read and approved the final review manuscript. Mingliang Zhang and Jie Yin conceived the article. Mingliang Zhang wrote the first version of the manuscript. Mingliang Zhang and Weimin Jiang made the outline and formatted the figures. Dingfu Xiao and Jie Yin oversaw and directed the entire work.

## Funding

This work was supported by the Key Research and Development Program of Hunan Province (2023NK2018), and earmarked Fund for China Agriculture Research System (CARS-37).

## Declaration of competing interest

The authors declare that they have no known competing financial interests or personal relationships that could have appeared to influence the work reported in this paper.

## References

[1] Khalifeh M., Santos R.D., Oskuee R.K., Badiee A., Aghaee-Bakhtiari S.H., Sahebkar A. (2023). A novel regulatory facet for hypertriglyceridemia: the role of microRNAs in the regulation of triglyceride-rich lipoprotein biosynthesis. Prog Lipid Res.

[2] Simha V. (2020). Management of hypertriglyceridemia. BMJ.

[3] Hansen S.E., Varbo A., Nordestgaard B.G., Langsted A. (2023). Hypertriglyceridemia-associated pancreatitis: new concepts and potential mechanisms. Clin Chem.

[4] Ruiz-García A., Arranz-Martínez E., López-Uriarte B., Rivera-Teijido M., Palacios-Martínez D., Dávila-Blázquez G.M. (2020). Prevalence of hypertriglyceridemia in adults and related cardiometabolic factors. SIMETAP-HTG study. Clínica e Investigación en Arteriosclerosis (English Edition).

[5] Pirillo A., Casula M., Olmastroni E., Norata G.D., Catapano A.L. (2021). Global epidemiology of dyslipidaemias. Nat Rev Cardiol.

[6] Mclelland G.L., Lopez-Osias M., Verzijl C.R.C., Ellenbroek B.D., Oliveira R.A., Boon N.J. (2023). Identification of an alternative triglyceride biosynthesis pathway. Nature.

[7] Ko C.-W., Qu J., Black D.D., Tso P. (2020). Regulation of intestinal lipid metabolism: current concepts and relevance to disease. Nat Rev Gastroenterol Hepatol.

[8] Li Y., Zhang Z., Han Q., Liu G., Yin Y., Yin J. (2025). Lactobacillus johnsonii-derived leucic acid promotes fatty acid absorption and deposition by targeting CD36. Sci China Life Sci.

[9] Boren J., Taskinen M.-R., Björnson E., Packard C.J. (2022). Metabolism of triglyceride-rich lipoproteins in health and dyslipidaemia. Nat Rev Cardiol.

[10] Jia B., Zou Y., Han X., Bae J.-W., Jeon C.O. (2023). Gut microbiome-mediated mechanisms for reducing cholesterol levels: implications for ameliorating cardiovascular disease. Trends Microbiol.

[11] Li G., Liu L., Lu T., Sui Y., Zhang C., Wang Y. (2023). Gut microbiota aggravates neutrophil extracellular traps-induced pancreatic injury in hypertriglyceridemic pancreatitis. Nat Commun.

[12] Xu H., Fang F., Wu K., Song J., Li Y., Lu X. (2023). Gut microbiota-bile acid crosstalk regulates murine lipid metabolism via the intestinal FXR-FGF19 axis in diet-induced humanized dyslipidemia. Microbiome.

[13] Flaig B., Garza R., Singh B., Hamamah S., Covasa M. (2023). Treatment of dyslipidemia through targeted therapy of gut microbiota. Nutrients.

[14] Wang H., Ma C., Li Y., Zhang L., Yang C., Zhao F. (2023). Probio-X relieves symptoms of hyperlipidemia by regulating patients’ gut microbiome, blood lipid metabolism, and Lifestyle habits. Microbiol Spectrum.

[15] Ahmad H.F., Mejia J.L.C., Krych L., Khakimov B., Kot W., Bechshøft R.L. (2020). IDDF2020-ABS-0174 Onset of hypertriglyceridemia in relation to dietary intake, gut microbiome and metabolomics signatures among home dwelling elderly. Gut.

[16] Miao G., Guo J., Lai P., Chen J., Zhou Z., Zhang W. (2022). Correction of hypertriglyceridemia by intestinal microbiota remodeling alleviates NASH and atherosclerosis in severe combined hyperlipidemia disease. Research Square.

[17] Kong L., Yu S., Gu L., Geng M., Zhang D., Cao H. (2022). Associations of typical antibiotic residues with elderly blood lipids and dyslipidemia in West Anhui, China. Ecotoxicol Environ Saf.

[18] Ni Y., Qian L., Siliceo S.L., Long X., Nychas E., Liu Y. (2023). Resistant starch decreases intrahepatic triglycerides in patients with NAFLD via gut microbiome alterations. Cell Metab.

[19] Wang Y., Ai Z., Xing X., Fan Y., Zhang Y., Nan B. (2024). The ameliorative effect of probiotics on diet-induced lipid metabolism disorders: a review. Crit Rev Food Sci Nutr.

[20] Murali S.K., Mansell T.J. (2024). Next generation probiotics: Engineering live biotherapeutics. Biotechnol Adv.

[21] O’Toole P.W., Marchesi J.R., Hill C. (2017). Next-generation probiotics: the spectrum from probiotics to live biotherapeutics. Nat Microbiol.

[22] Ahmad H.F., Castro Mejia J.L., Krych L., Khakimov B., Kot W., Bechshøft R.L. (2020). Gut Mycobiome dysbiosis is linked to hypertriglyceridemia among home Dwelling elderly Danes. BioRxiv.

[23] Hu X., Gong L., Zhou R., Han Z., Ji L., Zhang Y. (2021). Variations in gut microbiome are associated with prognosis of hypertriglyceridemia-associated acute pancreatitis. Biomolecules.

[24] Liu X., Tong X., Zou Y., Lin X., Zhao H., Tian L. (2022). Mendelian randomization analyses support causal relationships between blood metabolites and the gut microbiome. Nat Genet.

[25] Xiao Y., Zhao J., Zhang H., Zhai Q., Chen W. (2020). Mining Lactobacillus and Bifidobacterium for organisms with long-term gut colonization potential. Clin Nutr.

[26] Menjivar C., Pagella E., Biswas I. (2024). Akkermansia muciniphila. Trends Microbiol.

[27] Ioannou A., Berkhout M.D., Geerlings S.Y., Belzer C. (2024). Akkermansia muciniphila: biology, microbial ecology, host interactions and therapeutic potential. Nat Rev Microbiol.

[28] Plovier H., Everard A., Druart C., Depommier C., Van Hul M., Geurts L. (2017). A purified membrane protein from Akkermansia muciniphila or the pasteurized bacterium improves metabolism in obese and diabetic mice. Nat Med.

[29] Kim S., Lee Y., Kim Y., Seo Y., Lee H., Ha J. (2020). Akkermansia muciniphila prevents fatty liver disease, decreases serum triglycerides, and maintains gut homeostasis. Appl Environ Microbiol.

[30] Depommier C., Everard A., Druart C., Plovier H., Van Hul M., Vieira-Silva S. (2019). Supplementation with Akkermansia muciniphila in overweight and obese human volunteers: a proof-of-concept exploratory study. Nat Med.

[31] Yoon H.S., Cho C.H., Yun M.S., Jang S.J., You H.J., Kim J.-h. (2021). Akkermansia muciniphila secretes a glucagon-like peptide-1-inducing protein that improves glucose homeostasis and ameliorates metabolic disease in mice. Nat Microbiol.

[32] Gauffin Cano P., Santacruz A., Moya Á., Sanz Y. (2012). Bacteroides uniformis CECT 7771 ameliorates metabolic and immunological dysfunction in mice with high-fat-diet induced obesity. PLoS One.

[33] Xu M., Lan R., Qiao L., Lin X., Hu D., Zhang S. (2023). Bacteroides vulgatus ameliorates lipid metabolic disorders and modulates gut microbial composition in hyperlipidemic rats. Microbiol Spectrum.

[34] Sun C., Xiong X., Liu M., Liang Q., Zhao Q., Wei G. (2024). Bacteroides ovatus alleviates high-fat and high-cholesterol-induced nonalcoholic fatty liver disease via gut-liver axis. Biomed Pharmacother.

[35] Huang Y., Cao J., Zhu M., Wang Z., Jin Z., Xiong Z. (2024). Bacteroides fragilis aggravates high-fat diet-induced non-alcoholic fatty liver disease by regulating lipid metabolism and remodeling gut microbiota. Microbiol Spectrum.

[36] Stoeva M.K., Garcia-So J., Justice N., Myers J., Tyagi S., Nemchek M. (2021). Butyrate-producing human gut symbiont, Clostridium butyricum, and its role in health and disease. Gut Microbes.

[37] Liu J., Zhang S., Weng H. (2024). Effects of Clostridium butyricum and inulin supplementation on intestinal microbial composition in high-fat diet fed mice. Food Funct.

[38] Zhu W., Yan M., Cao H., Zhou J., Xu Z. (2022). Effects of Clostridium butyricum capsules combined with rosuvastatin on intestinal flora, lipid metabolism, liver function and inflammation in NAFLD patients. Cell Mol Biol.

[39] Mukherjee A., Lordan C., Ross R.P., Cotter P.D. (2020). Gut microbes from the phylogenetically diverse genus Eubacterium and their various contributions to gut health. Gut Microbes.

[40] Riggen-Bueno V., Del Toro-Arreola S., Baltazar-Díaz T.A., Vega-Magaña A.N., Peña-Rodríguez M., Castaño-Jiménez P.A. (2024). Intestinal dysbiosis in subjects with obesity from western mexico and its association with a proinflammatory profile and disturbances of folate (B9) and carbohydrate metabolism. Metabolites.

[41] Udayappan S., Manneras-Holm L., Chaplin-Scott A., Belzer C., Herrema H., Dallinga-Thie G.M. (2016). Oral treatment with Eubacterium hallii improves insulin sensitivity in db/db mice. npj Biofilms Microbiomes.

[42] Leylabadlo H.E., Ghotaslou R., Feizabadi M.M., Farajnia S., Moaddab S.Y., Ganbarov K. (2020). The critical role of Faecalibacterium prausnitzii in human health: an overview. Microb Pathog.

[43] Yang M., Wang J.-H., Shin J.-H., Lee D., Lee S.-N., Seo J.-G. (2023). Pharmaceutical efficacy of novel human-origin Faecalibacterium prausnitzii strains on high-fat-diet-induced obesity and associated metabolic disorders in mice. Front Endocrinol.

[44] Munukka E., Rintala A., Toivonen R., Nylund M., Yang B., Takanen A. (2017). Faecalibacterium prausnitzii treatment improves hepatic health and reduces adipose tissue inflammation in high-fat fed mice. ISME J.

[45] Hu W., Gao W., Liu Z., Fang Z., Wang H., Zhao J. (2022). Specific strains of faecalibacterium prausnitzii ameliorate nonalcoholic fatty liver disease in mice in association with gut microbiota regulation. Nutrients.

[46] Xuan W., Ou Y., Chen W., Huang L., Wen C., Huang G. (2023). Faecalibacterium prausnitzii improves lipid metabolism disorder and insulin resistance in type 2 diabetic mice. Br J Biomed Sci.

[47] Cui Y., Zhang L., Wang X., Yi Y., Shan Y., Liu B. (2022). Roles of intestinal Parabacteroides in human health and diseases. FEMS Microbiol Lett.

[48] Xu M., Lü L., Hu Y., Zhang M., Shen J., Liu C. (2024). Oligomeric procyanidins combined with Parabacteroides distasonis ameliorate high-fat diet-induced atherosclerosis by regulating lipid metabolism, inflammation reaction and bile acid metabolism in ApoE−/− mice. Food Sci Human Wellness.

[49] Sun Y., Nie Q., Zhang S., He H., Zuo S., Chen C. (2023). Parabacteroides distasonis ameliorates insulin resistance via activation of intestinal GPR109a. Nat Commun.

[50] Wang K., Liao M., Zhou N., Bao L., Ma K., Zheng Z. (2019). Parabacteroides distasonis alleviates obesity and metabolic dysfunctions via production of succinate and secondary bile acids. Cell Rep.

[51] Lai C.-H., Lin T.-L., Huang M.-Z., Li S.-W., Wu H.-Y., Chiu Y.-F. (2022). Gut commensal Parabacteroides goldsteinii mts01 alters gut microbiota composition and reduces cholesterol to mitigate Helicobacter pylori-induced pathogenesis. Front Immunol.

[52] Wu T.-R., Lin C.-S., Chang C.-J., Lin T.-L., Martel J., Ko Y.-F. (2019). Gut commensal Parabacteroides goldsteinii plays a predominant role in the anti-obesity effects of polysaccharides isolated from Hirsutella sinensis. Gut.

[53] Pabst O., Hornef M.W., Schaap F.G., Cerovic V., Clavel T., Bruns T. (2023). Gut–liver axis: barriers and functional circuits. Nat Rev Gastroenterol Hepatol.

[54] Ghosh S., Whitley C.S., Haribabu B., Jala V.R. (2021). Regulation of intestinal barrier function by microbial metabolites. Cell Mol Gastroenterol Hepatol.

[55] Tan X., Liu Y., Long J., Chen S., Liao G., Wu S. (2019). Trimethylamine N‐oxide aggravates liver steatosis through modulation of bile acid metabolism and inhibition of farnesoid X receptor signaling in nonalcoholic fatty liver disease. Mol Nutr Food Res.

[56] Nikolaeva S., Fock E., Parnova R. (2022). Lipopolysaccharide stimulates triglyceride accumulation and lipid droplet biogenesis in PC12 cells: the role of carnitine palmitoyltransferase 1 down-regulation and suppression of fatty acid oxidation. J Evol Biochem Physiol.

[57] Shimizu H., Masujima Y., Ushiroda C., Mizushima R., Taira S., Ohue-Kitano R. (2019). Dietary short-chain fatty acid intake improves the hepatic metabolic condition via FFAR3. Sci Rep.

[58] Watanabe M., Houten S.M., Wang L., Moschetta A., Mangelsdorf D.J., Heyman R.A. (2004). Bile acids lower triglyceride levels via a pathway involving FXR, SHP, and SREBP-1c. J Clin Invest.

[59] Mann E.R., Lam Y.K., Uhlig H.H. (2024). Short-chain fatty acids: linking diet, the microbiome and immunity. Nat Rev Immunol.

[60] Zoetendal E.G., Raes J., Van Den Bogert B., Arumugam M., Booijink C.C., Troost F.J. (2012). The human small intestinal microbiota is driven by rapid uptake and conversion of simple carbohydrates. ISME J.

[61] Campos-Perez W., Martinez-Lopez E. (2021). Effects of short chain fatty acids on metabolic and inflammatory processes in human health. Biochim et Biophys Acta (BBA)-Mol Cell Biol Lipids.

[62] Tan H., Zhai Q., Chen W. (2019). Investigations of Bacteroides spp. towards next-generation probiotics. Food Res Int.

[63] Lu Y., Fan C., Li P., Lu Y., Chang X., Qi K. (2016). Short chain fatty acids prevent high-fat-diet-induced obesity in mice by regulating G protein-coupled receptors and gut microbiota. Sci Rep.

[64] Li F., Tai L., Sun X., Lv Z., Tang W., Wang T. (2024). Molecular recognition and activation mechanism of short-chain fatty acid receptors FFAR2/3. Cell Res.

[65] Ikeda T., Nishida A., Yamano M., Kimura I. (2022). Short-chain fatty acid receptors and gut microbiota as therapeutic targets in metabolic, immune, and neurological diseases. Pharmacol Ther.

[66] Zhang B., Liu H., Liu M., Yue Z., Liu L., Fuchang L. (2022). Exogenous butyrate regulates lipid metabolism through GPR41-ERK-AMPK pathway in rabbits. Ital J Anim Sci.

[67] Zaibi M.S., Stocker C.J., O’Dowd J., Davies A., Bellahcene M., Cawthorne M.A. (2010). Roles of GPR41 and GPR43 in leptin secretory responses of murine adipocytes to short chain fatty acids. FEBS Lett.

[68] Stern J.H., Rutkowski J.M., Scherer P.E. (2016). Adiponectin, leptin, and fatty acids in the maintenance of metabolic homeostasis through adipose tissue crosstalk. Cell Metab.

[69] Jiao A., Yu B., He J., Yu J., Zheng P., Luo Y. (2021). Sodium acetate, propionate, and butyrate reduce fat accumulation in mice via modulating appetite and relevant genes. Nutrition.

[70] Frampton J., Murphy K.G., Frost G., Chambers E.S. (2020). Short-chain fatty acids as potential regulators of skeletal muscle metabolism and function. Nat Metab.

[71] Li J., Zhao J., Tian C., Dong L., Kang Z., Wang J. (2024). Mechanisms of regulation of glycolipid metabolism by natural compounds in plants: effects on short-chain fatty acids. Nutr Metab.

[72] Hu G.-X., Chen G.-R., Xu H., Ge R.-S., Lin J. (2010). Activation of the AMP activated protein kinase by short-chain fatty acids is the main mechanism underlying the beneficial effect of a high fiber diet on the metabolic syndrome. Med Hypotheses.

[73] Herzig S., Shaw R.J. (2018). AMPK: guardian of metabolism and mitochondrial homeostasis. Nat Rev Mol Cell Biol.

[74] He J., Zhang P., Shen L., Niu L., Tan Y., Chen L. (2020). Short-chain fatty acids and their association with signalling pathways in inflammation, glucose and lipid metabolism. Int J Mol Sci.

[75] Henriksen B.S., Curtis M.E., Fillmore N., Cardon B.R., Thomson D.M., Hancock C.R. (2013). The effects of chronic AMPK activation on hepatic triglyceride accumulation and glycerol 3-phosphate acyltransferase activity with high fat feeding. Diabetol Metab Syndr.

[76] Foretz M., Even P.C., Viollet B. (2018). AMPK activation reduces hepatic lipid content by increasing fat oxidation in vivo. Int J Mol Sci.

[77] Garcia D., Shaw R.J. (2017). AMPK: mechanisms of cellular energy sensing and restoration of metabolic balance. Mol Cell.

[78] Guo L., Kang J.S., Park Y.H., Je B.I., Lee Y.J., Kang N.J. (2020). S-petasin inhibits lipid accumulation in oleic acid-induced HepG2 cells through activation of the AMPK signaling pathway. Food Funct.

[79] Wang Q., Sun J., Liu M., Zhou Y., Zhang L., Li Y. (2021). The new role of AMP-activated protein kinase in regulating fat metabolism and energy expenditure in adipose tissue. Biomolecules.

[80] Li X., Chen H., Guan Y., Li X., Lei L., Liu J. (2013). Acetic acid activates the AMP-activated protein kinase signaling pathway to regulate lipid metabolism in bovine hepatocytes. PLoS One.

[81] Shimano H., Sato R. (2017). SREBP-regulated lipid metabolism: convergent physiology — divergent pathophysiology. Nat Rev Endocrinol.

[82] Fushimi T., Suruga K., Oshima Y., Fukiharu M., Tsukamoto Y., Goda T. (2006). Dietary acetic acid reduces serum cholesterol and triacylglycerols in rats fed a cholesterol-rich diet. Br J Nutr.

[83] Jiao A., Yu B., He J., Yu J., Zheng P., Luo Y. (2020). Short chain fatty acids could prevent fat deposition in pigs via regulating related hormones and genes. Food Funct.

[84] Awazawa M., Ueki K., Inabe K., Yamauchi T., Kaneko K., Okazaki Y. (2009). Adiponectin suppresses hepatic SREBP1c expression in an AdipoR1/LKB1/AMPK dependent pathway. Biochem Biophys Res Commun.

[85] Kim S.-J., Tang T., Abbott M., Viscarra J.A., Wang Y., Sul H.S. (2016). AMPK phosphorylates desnutrin/ATGL and hormone-sensitive lipase to regulate lipolysis and fatty acid oxidation within adipose tissue. Mol Cell Biol.

[86] Tang T., Song J., Li J., Wang H., Zhang Y., Suo H. (2020). A synbiotic consisting of Lactobacillus plantarum S58 and hull-less barley β-glucan ameliorates lipid accumulation in mice fed with a high-fat diet by activating AMPK signaling and modulating the gut microbiota. Carbohydr Polym.

[87] Li J., Wu K., Zhong Y., Kuang J., Huang N., Guo X. (2023). Si–Ni-SAN ameliorates obesity through AKT/AMPK/HSL pathway-mediated lipolysis: Network pharmacology and experimental validation. J Ethnopharmacol.

[88] Zhang W., Kong L., Zhong Z., Lin L., Li J., Zheng G. (2023). Short chain fatty acids increase fat oxidation and promote browning through β3-adrenergic receptor/AMP-activated protein kinase α signaling pathway in 3T3-L1 adipocytes. J Funct Foods.

[89] Ding L., Huwyler F., Long F., Yang W., Binz J., Wernlé K. (2024). Glucose controls lipolysis through Golgi PtdIns4P-mediated regulation of ATGL. Nat Cell Biol.

[90] Song Z., Feng S., Zhou X., Song Z., Li J., Li P. (2023). Taxonomic identification of bile salt hydrolase‐encoding lactobacilli: Modulation of the enterohepatic bile acid profile. iMeta.

[91] Song Z., Cai Y., Lao X., Wang X., Lin X., Cui Y. (2019). Taxonomic profiling and populational patterns of bacterial bile salt hydrolase (BSH) genes based on worldwide human gut microbiome. Microbiome.

[92] Liu Y., Zheng S., Cui J., Guo T., Zhang J. (2021). Effect of bile salt hydrolase-active Lactobacillus plantarum Y15 on high cholesterol diet induced hypercholesterolemic mice. CyTA-J Food.

[93] Joyce S.A., Macsharry J., Casey P.G., Kinsella M., Murphy E.F., Shanahan F. (2014). Regulation of host weight gain and lipid metabolism by bacterial bile acid modification in the gut. PNAS.

[94] Collins S.L., Stine J.G., Bisanz J.E., Okafor C.D., Patterson A.D. (2023). Bile acids and the gut microbiota: metabolic interactions and impacts on disease. Nat Rev Microbiol.

[95] Tang J., Xu W., Yu Y., Yin S., Ye B.-C., Zhou Y. (2024). The role of the gut microbial metabolism of sterols and bile acids in human health. Biochimie.

[96] Perino A., Schoonjans K. (2022). Metabolic messengers: bile acids. Nat Metab.

[97] Zheng D., Zhang H., Zheng X., Zhao A., Jia W. (2024). Novel microbial modifications of bile acids and their functional implications. iMeta.

[98] Chiang J.Y., Ferrell J.M. (2019). Bile acids as metabolic regulators and nutrient sensors. Annu Rev Nutr.

[99] Wen X., Feng X., Xin F., An R., Huang H., Mao L. (2024). B. vulgatus ameliorates high-fat diet-induced obesity through modulating intestinal serotonin synthesis and lipid absorption in mice. Gut Microbes.

[100] Clifford B.L., Sedgeman L.R., Williams K.J., Morand P., Vallim T.Q.D.A. (2021). FXR activation protects against NAFLD via bile-acid-dependent reductions in lipid absorption. Cell Metab.

[101] Guan B., Tong J., Hao H., Yang Z., Chen K., Xu H. (2022). Bile acid coordinates microbiota homeostasis and systemic immunometabolism in cardiometabolic diseases. Acta Pharm Sin B.

[102] Xu S., Jia P., Fang Y., Jin J., Sun Z., Zhou W. (2022). Nuclear farnesoid X receptor attenuates acute kidney injury through fatty acid oxidation. Kidney Int.

[103] Zhong J., He X., Gao X., Liu Q., Zhao Y., Hong Y. (2023). Hyodeoxycholic acid ameliorates nonalcoholic fatty liver disease by inhibiting RAN-mediated PPARα nucleus-cytoplasm shuttling. Nat Commun.

[104] Packard C.J., Pirillo A., Tsimikas S., Ference B.A., Catapano A.L. (2023). Exploring apolipoprotein C-III: pathophysiological and pharmacological relevance. Cardiovasc Res.

[105] Urizar N.L., Dowhan D.H., Moore D.D. (2000). The farnesoid X-activated receptor mediates bile acid activation of phospholipid transfer protein gene expression. J Biol Chem.

[106] Mak P.A. (2002). Identification of PLTP as an LXR target gene and apoE as an FXR target gene reveals overlapping targets for the two nuclear receptors. J Lipid Res.

[107] Lun W., Yan Q., Guo X., Zhou M., Bai Y., He J. (2024). Mechanism of action of the bile acid receptor TGR5 in obesity. Acta Pharm Sin B.

[108] Wang H., Wang J., Cui H., Fan C., Xue Y., Liu H. (2024). Inhibition of fatty acid uptake by TGR5 prevents diabetic cardiomyopathy. Nat Metab.

[109] Xiao C., Bandsma R.H.J., Dash S., Szeto L., Lewis G.F. (2012). Exenatide, a glucagon-like peptide-1 receptor agonist, acutely inhibits intestinal lipoprotein production in healthy humans. Arterioscler Thromb Vasc Biol.

[110] Parlevliet E.T., Yanan W., Geerling J.J., Schr?Der-Van D.E.J.P., Kristen P., Karyn O.N. (2012). GLP-1 receptor activation inhibits VLDL production and reverses hepatic steatosis by decreasing hepatic lipogenesis in high-fat-fed APOE*3-leiden mice. PLoS One.

[111] Wang X., Liu J., Li C., Zhao M., Liu L., Guan Q. (2018). Impaired secretion of active GLP-1 in patients with hypertriglyceridaemia: a novel lipotoxicity paradigm?. Diabetes Metab Res Rev.

[112] Fan M., Wang Y., Jin L., Fang Z., Peng J., Tu J. (2022). Bile acid-mediated activation of brown fat protects from alcohol-induced steatosis and liver injury in mice. Cell Mol Gastroenterol Hepatol.

[113] Li Y., Zhu L., Cai M.-X., Wang Z.-L., Zhuang M., Tan C.-Y. (2023). TGR5 supresses cGAS/STING pathway by inhibiting GRP75-mediated endoplasmic reticulum-mitochondrial coupling in diabetic retinopathy. Cell Death Dis.

[114] Raposeiras-Roubin S., Rosselló X., Oliva B., Fernández-Friera L., Mendiguren J.M., Andrés V. (2021). Triglycerides and residual atherosclerotic risk. J Am Coll Cardiol.

[115] Pols T.W., Nomura M., Harach T., Sasso G.L., Oosterveer M.H., Thomas C. (2011). TGR5 activation inhibits atherosclerosis by reducing macrophage inflammation and lipid loading. Cell Metab.

[116] Afshar N., Amini K., Mohajerani H., Saki S. (2024). Evaluation of probiotic bifidobacteria strains from Iranian traditional dairy products for their anti-hyperlipidemic potential. Folia Microbiol.

[117] Li K., Gu Q., Yang W., Yu X. (2023). In vitro screening and probiotic evaluation of anti-obesity and antioxidant lactic acid bacteria. Food Biosci.

[118] Ewell M., Hind J., Jones-Meehan J., Jones W. (2001).

[119] Teichman E.M., O’Riordan K.J., Gahan C.G.M., Dinan T.G., Cryan J.F. (2020). When rhythms meet the blues: circadian interactions with the microbiota-gut-brain axis. Cell Metab.

[120] Zhang M., Zhou C., Li X., Li H., Han Q., Chen Z. (2025). Interactions between gut microbiota, host circadian rhythms, and metabolic diseases. Adv Nutr.

